# Auto immune haemolytic anaemia as part of Nevirapine induced DRESS syndrome

**DOI:** 10.1186/1471-2334-14-S3-P68

**Published:** 2014-05-27

**Authors:** Aravind Reghukumar, Athul Gurudas, VS Kiran Kumar, Ranjani Ravi

**Affiliations:** 1 Department of Infectious Diseases, Medical College Hospital, Thiruvananthapuram, India; 2 Cosmopolitan Hospital, Thiruvananthapuram, India

## Background

Drug rash with eosinophilia and systemic symptoms (DRESS) syndrome is an idiosyncratic hypersensitivity reaction to drugs. The diagnosis involves 3 criteria; 1)Drug induced skin eruption 2) Absolute eosinophil count (AEC) >1500/dL or atypical lymphocytosis 3) One of the following enlarged lympnodes atleast 2cm in diameter, hepatitis, interstitial nephritis, interstitial lung disease or myocarditis. Nevirapine and abacavir are known to produce DRESS syndrome. Autoimmune hemolytic anemia [AIHA] as a part of nevirapine induced DRESS syndrome is extremely rare. To the best of our knowledge there are no cases reported so far. We are reporting two cases of the same.

## Case report

45 year old male, with CD4 190 was started on zidovudine, lamivudine, nevirapine and cotrimoxazole. Two weeks later, he presented with fever, generalized maculopapular pruritic rash and was found to have pallor, icterus, multiple cervical lymphnodes and hepatosplenomegaly. All drugs were withheld suspecting hypersensitivity. Routine investigations showed progressive anaemia, eosinophilia, indirect hyperbilirubinemia with transaminitis and interstitial nephritis. Corrected reticulocyte count was 3%. Serum LDH was 620. Peripheral smear was suggestive of extravascular haemolysis. Direct Coombs test was positive. The patient was diagnosed to have DRESS syndrome with AIHA. Steroids were started with which he showed dramatic improvement. The patient tolerated zidovudine, lamivudine and cotrimoxazole on reintroduction thereby identifying the culprit drug as nevirapine.

A second case involved a 36 year old lady with CD4 110 who presented similarly, 3 weeks following initiation of HAART. She also improved with steroids. Nevirapine was again identified as the causative drug.

